# Dietary Strategies and Nutritional Management in Patients Receiving GLP‐1 and Dual GIP/GLP‐1 Receptor Agonists as Adjuncts to Lifestyle Interventions: A Systematic Review of Randomised Clinical Trials

**DOI:** 10.1111/dom.70779

**Published:** 2026-04-27

**Authors:** Rayanne Santos de Paulo, Dandara Baia Bonifácio, Matheus Henrique Lana de Carvalho, Josefina Bressan

**Affiliations:** ^1^ Department of Nutrition and Health Universidade Federal de Viçosa Viçosa Brazil

**Keywords:** diet, gastrointestinal symptoms, GIP/GLP‐1 receptor agonists, lean mass, nutrition, obesity, tirzepatide

## Abstract

**Background:**

Glucagon‐like peptide‐1 receptor agonists (GLP‐1 RAs) and dual GIP/GLP‐1 RAs are widely used to manage obesity, prediabetes and type 2 diabetes, typically in combination with lifestyle interventions. Their nutritional implications, however, remain unclear. This systematic review summarised evidence from randomised clinical trials investigating dietary strategies and nutritional management in individuals treated with these medications.

**Methods:**

The review followed PRISMA guidelines and was registered in PROSPERO (CRD420251181076). Searches were conducted in PubMed, Embase, Scopus and Web of Science. Eligible studies were randomised clinical trials evaluating adults receiving GLP‐1 or dual GIP/GLP‐1 agonists in conjunction with lifestyle or dietary guidance. Outcomes included gastrointestinal symptoms, muscle and bone health and nutritional adequacy.

**Results:**

Sixteen trials involving 7096 participants were included. GLP‐1 and dual agonists consistently increased gastrointestinal symptoms such as nausea, diarrhoea, constipation and vomiting. These effects were often dose‐related and occurred despite background lifestyle or dietary interventions. Lean mass was generally preserved, with reductions proportional to overall weight loss. No study directly assessed bone health, and none reported clinically relevant nutritional deficiencies. Most trials presented a low risk of bias.

**Conclusion:**

GLP‐1 and dual GIP/GLP‐1 agonists frequently cause gastrointestinal discomfort but tend to preserve lean mass during weight loss when used alongside lifestyle interventions. Although dietary guidance is commonly provided, evidence on optimal nutritional approaches remains limited. Further trials are needed to clarify protein requirements, assess musculoskeletal outcomes and establish evidence‐based dietary recommendations to support combined treatment strategies.

## 1 Introduction

Obesity and type 2 diabetes mellitus (T2DM) frequently coexist due to shared pathophysiological mechanisms, including chronic low‐grade inflammation, insulin resistance and disturbances in protein and lipid metabolism [1, 2]. The global burden of these conditions continues to rise, with an estimated 2.5 billion adults classified as overweight in 2022, including 890 million with obesity, alongside a substantial and growing prevalence among children and adolescents [3]. Diabetes currently affects ~537 million adults worldwide, with projections reaching 783 million by 2045 [4]. Both obesity and T2DM markedly elevate the risk of adverse outcomes ranging from metabolic dysfunction and cardiovascular complications to physical frailty, sarcopenia, poorer quality of life and higher mortality rates [5, 6]. Lifestyle intervention, including dietary modification, increased physical activity and behavioural counselling, remains the cornerstone of obesity and T2DM management. Clinical guidelines consistently recommend that these strategies be maintained throughout treatment, even when pharmacological therapies are introduced [7]. Accordingly, pharmacotherapy is typically implemented as an adjunct to background lifestyle interventions rather than as a standalone approach.

Among available pharmacological options, glucagon‐like peptide‐1 receptor agonists (GLP‐1 RAs) and dual glucose‐dependent insulinotropic polypeptide (GIP) and glucagon‐like peptide‐1 receptor agonists (GIP/GLP‐1 RAs) have emerged as particularly effective for improving glycaemic control and inducing clinically meaningful weight loss, contributing to their rapid expansion in clinical practice [8, 9]. These agents act through multiple mechanisms, including enhancement of glucose‐dependent insulin secretion, suppression of glucagon release and modulation of central appetite‐regulating pathways in the hindbrain, hypothalamus and mesolimbic system [10, 11]. As a result, they reduce appetite, increase satiety and lower overall energy intake [12, 13].

While these mechanisms promote effective weight loss, they also lead to significant reductions in energy intake. They may influence food choices, often decreasing the preference for high fat, energy‐dense foods [12]. However, these changes raise important nutritional concerns. Reduced dietary intake can compromise micronutrient adequacy, and insufficient protein intake may contribute to lean mass loss and impair muscle health [14]. In addition, gastrointestinal adverse events commonly observed during treatment may further influence dietary intake and food tolerance [15]. Despite the clear metabolic benefits and emerging evidence of cardiometabolic and renal protection [16], the extent to which GLP‐1–based therapies affect nutrient intake, meal composition and overall diet quality remains insufficiently understood [17, 18].

These considerations highlight the critical role of nutritional management in optimising GLP‐1 and GIP/GLP‐1 RAs therapy. Strategies such as gradual reduction of meal volume, adequate daily protein distribution, preference for fibre‐rich and low‐glycaemic index foods and maintaining hydration may enhance treatment tolerability, support satiety and help preserve muscle mass during weight loss [7, 19, 20]. Nonetheless, clinical recommendations remain largely empirical, and there is no consensus on the optimal dietary approach to accompany these medications.

Given that pharmacological treatment is typically delivered in conjunction with background lifestyle and dietary recommendations, understanding the nutritional context in which these therapies are applied is essential. Nutritional strategies such as adequate protein distribution, selection of fibre‐rich foods and individualised dietary adjustments may play a key role in optimising treatment tolerability and preserving lean mass during weight loss. However, current recommendations are largely empirical, and there is no clear consensus on the most effective dietary approaches to accompany these therapies.

Therefore, this systematic review aims to synthesise evidence from randomised controlled trials evaluating GLP‐1 and GIP/GLP‐1 receptor agonists as adjuncts to background lifestyle and nutritional interventions. The review focuses on characterising the dietary context in which these therapies are implemented, examining gastrointestinal adverse events, changes in muscle mass and broader aspects of nutritional management to inform clinical practice and identify existing gaps in the literature.

## 2 Methods

### 2.1 Protocol and Registration

This systematic review was conducted following the “Preferred Reporting Items for Systematic Reviews and Meta‐Analyses” (PRISMA) guidelines [21] and was prospectively registered in the PROSPERO database (registration ID: CRD420251181076; https://www.crd.york.ac.uk/PROSPERO/view/CRD420251181076).

### 2.2 Search Strategy

The research question that guided this systematic review was: “What are the reported nutritional considerations and outcomes in individuals with excess body weight, obesity, prediabetes, or diabetes treated with GLP‐1 and dual GIP/GLP‐1 receptor agonists in the context of background lifestyle and dietary interventions, and how do these relate to gastrointestinal adverse events, muscle and bone health and nutritional status?” To address this question and determine study eligibility, the PICOS framework (population, intervention, comparison, outcomes and study design) was applied, as presented in Table 1.

**TABLE 1:** Criteria for study selection based on the PICOS framework.

Parameter	Inclusion criteria
Population	Individuals with overweight/obesity, prediabetes, or diabetes treated with GLP‐1 and dual GIP/GLP‐1 RA Individuals with overweight/obesity, prediabetes or diabetes receiving treatment with GLP‐1 and dual GIP/GLP‐1 receptor agonists
Intervention	Dietary strategies and nutritional management are used as primary or secondary approaches Pharmacological treatment with GLP‐1 and dual GIP/GLP‐1 receptor agonists administered in conjunction with background lifestyle and dietary interventions
Comparison	With or without a comparator/control group for nutritional intervention Control groups receiving background lifestyle interventions, with or without pharmacological treatment.
Outcomes	Gastrointestinal effects, muscle and bone health and nutritional deficiencies
Study design	Randomised clinical trials

### 2.3 Eligibility Criteria

Based on the PICOS criteria, the inclusion parameters were defined as follows: (1) randomised clinical trials; (2) individuals with excess body weight, obesity, prediabetes, or diabetes receiving treatment with GLP‐1 and dual GIP/GLP‐1 receptor agonists; (3) pharmacological interventions administered in conjunction with lifestyle and dietary recommendations; and (4) studies including control or comparator group receiving background lifestyle interventions with or without pharmacological treatment. The primary outcomes were gastrointestinal effects (e.g., nausea, constipation, reflux and related symptoms); muscle and bone health (including muscle mass loss, sarcopenia and reduced bone mineral density); and nutritional deficiencies (e.g., vitamins, minerals and proteins).

The exclusion criteria were defined as follows: (1) studies not conducted in the context of combined pharmacological and lifestyle interventions; (2) articles that did not investigate the primary outcomes of interest; (3) research focused on other diseases, such as cancer; and (4) non‐original publications, such as case reports, reviews, book chapters, theses, dissertations, conference abstracts or proceedings, clinical trial registrations and studies conducted in animals or under in vitro conditions.

### 2.4 Selection of Studies and Data Collection Strategies

To develop the search strategy, keywords were defined using the PICOS framework and Medical Subject Headings (MeSH) terms, along with their corresponding English synonyms. These terms were combined using the Boolean operators AND, OR and NOT, with no restrictions regarding language or publication date, as presented in the Supporting Information. The databases consulted were MEDLINE/PubMed, Embase, Scopus and Web of Science.

All references identified during the search were imported into Rayyan (https://www.rayyan.ai/) for duplicate detection and to organise the screening process [22]. Two independent reviewers (RSP and MHLC) screened the titles and abstracts according to the predefined eligibility criteria. In cases of disagreement, a third investigator (DBB) was consulted to reach a final decision. Articles considered potentially relevant were then assessed in full text, also independently, to confirm their inclusion.

Data extraction was carried out by two independent authors (RSP and DBB) to collect key information, including study design, intervention characteristics, sample details, assessed outcomes and main findings. Subsequently, the extracted data were jointly reviewed, and any discrepancies were discussed and resolved. The articles were organised by year of publication and type of GLP‐1 receptor agonist used.

### 2.5 Risk‐of‐Bias Assessment

The risk of bias for each included study was independently assessed by two authors (RSP and DBB) using the Joanna Briggs Institute (JBI) Reviewer's Manual as a guide. Discrepancies between reviewers were resolved through discussion or consultation with a third author (MHLC). This evaluation aimed to determine the methodological rigour of each trial and the extent to which potential biases were addressed in the study design, conduct and analysis. Thirteen specific questions related to randomised clinical trials were applied to every study included in the systematic review. Responses to these questions were categorised as “low”, “high”, or “unclear” according to the predefined criteria.

## 3 Results

### 3.1 Characteristics and Selection of the Studies

**FIGURE 1:**
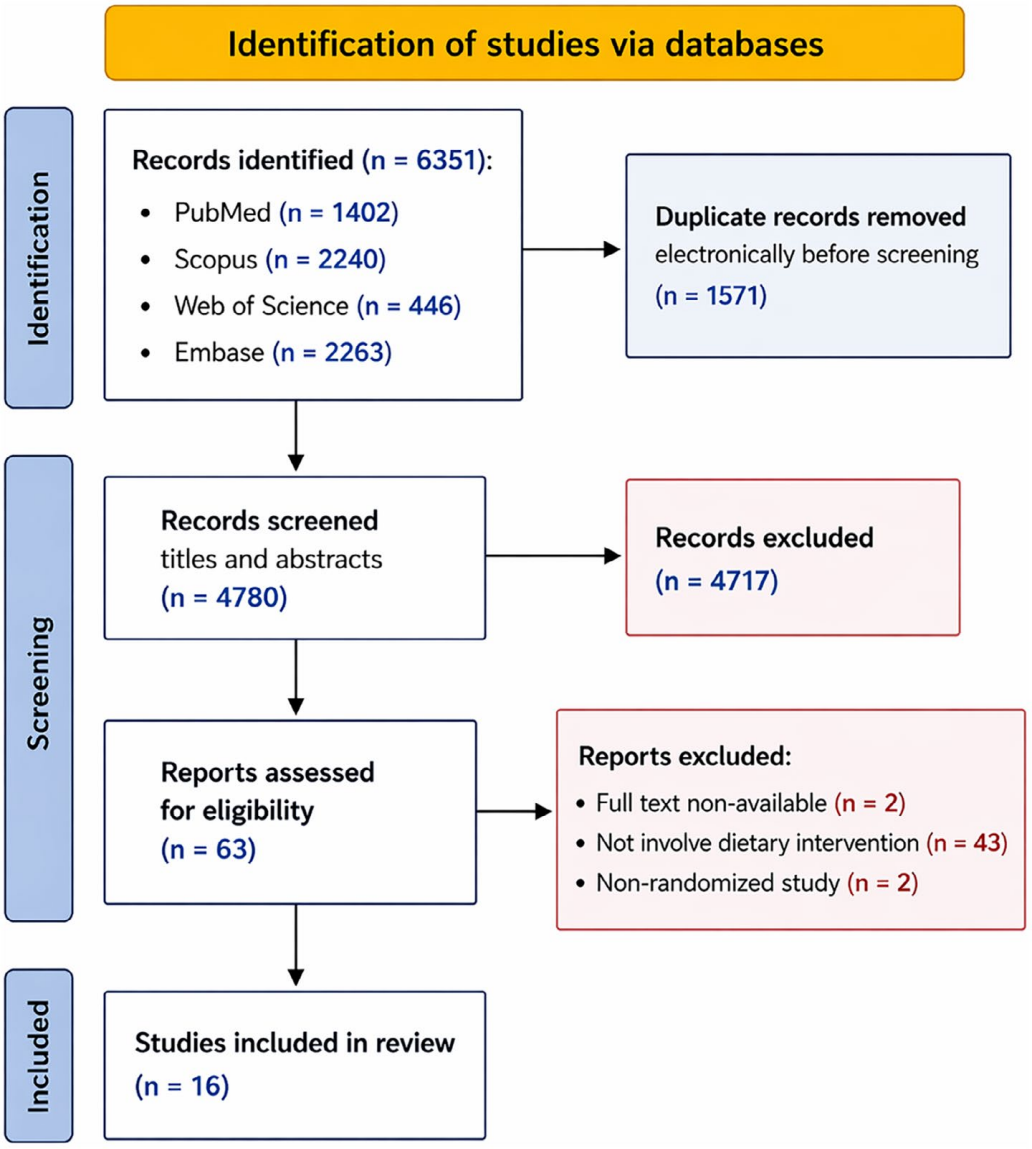
PRISMA flow diagram for systematic reviews.

A total of 6351 citations were retrieved from the PubMed/MEDLINE, Web of Science, Embase and Scopus databases from October 2025. Of the citations retrieved from the databases, 4780 titles remained after duplicates were removed using the Rayyan QCRY software. During screening of titles and abstracts, 4717 records were excluded based on the initial exclusion criteria. Of the remaining 63 articles, 10 were unanimously included by the first two reviewers (RSP and DBB). The other 53 presented disagreements, which were resolved by a third reviewer (MHLC), who decided to exclude 43 and include 8. However, we cannot access two of the included studies, so they are excluded. Therefore, this systematic review included 16 randomised clinical trial articles (Figure 1).

A total of 16 randomised controlled trials were included, comprising 7096 participants with sample sizes ranging from 40 to 3723, and all adults aged 18 to 75 years with overweight, obesity, prediabetes, or type 2 diabetes. Within the analysed studies, the interventions evaluated GLP‐1 receptor agonists (liraglutide, semaglutide, dulaglutide, efpeglenatide, albiglutide) and dual GIP/GLP‐1 agonists (tirzepatide), compared with placebo.

All studies combined pharmacological treatment received weekly or daily subcutaneous doses of the agonists, often with dose escalation during the initial weeks. This was accompanied by dietary or lifestyle guidance that ranged from standardised nutritional advice and calorie‐restricted diets (typically a 500 kcal/day deficit) to structured behavioural counselling, weekly or biweekly nutrition consultations, and supervised physical activity. Intervention durations ranged from 12 to 88 weeks, with one trial totalling 3 years.

Most trials assessed gastrointestinal adverse events, including nausea, vomiting, diarrhoea, constipation, abdominal pain, dyspepsia, belching and abdominal fullness. Some studies also examined muscle mass.

### 3.2 Evaluated Outcomes

#### 3.2.1 Gastrointestinal Effects

The combination of adjunct lifestyle interventions did not attenuate these adverse effects; even with a nutritional intervention, there was no comparison with a group that did not receive one. Thus, Sandsdal et al. [23] demonstrated that gastrointestinal events remained elevated regardless of supervised exercise participation, with liraglutide groups showing an incidence of 71%–86%, compared with 45% in the placebo group. Zhang et al. [24] reported that dulaglutide produced gastrointestinal symptoms in 37.1% of participants, whereas no gastrointestinal events occurred in the caloric‐restriction control group.

Gastrointestinal adverse events were the most frequently reported outcomes across the included trials, with nausea, diarrhoea, vomiting and constipation more common in GLP‐1 agonist groups than in placebo groups. Aronne et al. [25] described high rates of gastrointestinal symptoms in the initial phase of tirzepatide treatment, including nausea (35.5%), diarrhoea (21.1%), constipation (20.7%) and vomiting (16.3%), although these events declined during the double blind period. Similar findings were observed by Frias et al. [26], who reported gastrointestinal effects in 44.1% to 70.4% of participants receiving orforglipron across dose groups, compared with 18.2% in the placebo group, and in 34% of those treated with dulaglutide.

Semaglutide also led to increased gastrointestinal symptoms. Gu et al. [27] reported higher rates of diarrhoea (28.2% vs. 6.7%) and nausea (20.5% vs. 4.8%) with semaglutide 2.4 mg compared with placebo. A dose–response effect was evident in Lingvay et al. [28], with gastrointestinal events occurring in 53.1% of individuals receiving 7.2 mg, 51.5% of those receiving 2.4 mg and 25.5% of those receiving a placebo. Consistent findings were also reported by Neil et al. [29], who found that gastrointestinal disturbances affected 62%–82% of participants treated with semaglutide, depending on dose, compared with 75% in both the liraglutide and placebo groups.

Trials evaluating liraglutide showed a consistent pattern of increased gastrointestinal symptoms compared with placebo. Halawi et al. [30] found nausea in 63.15% of participants receiving liraglutide compared with 19% in the placebo group. Kim et al. [31] similarly reported nausea rates of 67% versus 26%. Nexøe‐Larsen et al. [32] also observed a substantial difference, with gastrointestinal events occurring in 88.5% of individuals treated with liraglutide compared with 34.6% in the placebo group. Larger trials reinforced these findings, Pi‐Sunyer et al. [33] documented higher frequencies of nausea, diarrhoea, constipation and vomiting in the liraglutide group than in the placebo group.

Other GLP‐1 agonists also demonstrated gastrointestinal adverse‐event profiles consistent with this drug class. In Pratley et al. [34], efpeglenatide across dosing schedules was associated with higher frequencies of gastrointestinal symptoms than placebo. Nausea ranged from 54.2% to 62.1%, compared with 18.3% in the placebo group. Vomiting occurred in 22% to 32.8% of participants versus 6.7% in the placebo and diarrhoea ranged from 20.3% to 27.6%, slightly above the 20% reported in the placebo. Dyspepsia increased from 15.3% to 27.1%, compared with 3.3% in the placebo group and constipation rose from 16.9% to 20.7%, exceeding the 8.3% observed in the placebo group.

In Nauck et al. [35], albiglutide was associated with mild elevations in gastrointestinal events compared with placebo. Nausea occurred in 9.1% to 9.9% of participants receiving albiglutide compared with 7.9% in the placebo group, and diarrhoea ranged from 9.9% to 13.1%, slightly above the 11.9% reported in the placebo group. Vomiting remained infrequent but was still higher with albiglutide, occurring in up to 3% of participants compared with 1% in placebo. Other symptoms, including reflux and dyspepsia, showed minor variations but either approximated or slightly exceeded placebo values.

Additional studies showed moderate increases in gastrointestinal symptoms. Kimura et al. [36] identified higher symptom rates with semaglutide than with dulaglutide, and Lundkvist et al. [37] reported gastrointestinal events in 64% of individuals receiving dapagliflozin combined with exenatide compared with 40% in the placebo group.

Overall, despite the diversity of populations studied, including individuals with obesity, overweight, type 2 diabetes, prediabetes and polycystic ovarian syndrome (PCOS), gastrointestinal adverse events followed a consistent pattern across GLP‐1 agonists. Higher doses were associated with increased frequencies of nausea, diarrhoea and vomiting and placebo groups consistently showed substantially lower rates.

### 3.3 Muscular Effects

Assessments related to muscle health were limited across the included trials, yet the available findings consistently indicate that lean mass was largely preserved during treatment. In the three studies that evaluated body composition, lean mass remained relatively stable, suggesting that GLP‐1‐based therapies do not compromise muscle preservation, even in the context of weight loss.

Kadouh et al. [38] observed a lean mass reduction of 1.3 kg with liraglutide compared with −0.65 kg in the placebo group. Although a slight decrease occurred, this pattern likely reflects the expected consequence of overall weight reduction rather than a specific adverse effect of the drug, indicating that muscle loss was minimal.

Similarly, Zhang et al. [24] found no significant differences in lean mass percentage or absolute lean mass between dulaglutide and caloric restriction alone. This demonstrates that muscle tissue was maintained despite weight reduction, supporting the idea of preserved lean mass with GLP‐1 therapy. Lundkvist et al. [37], evaluating dapagliflozin plus exenatide, also reported no significant changes in lean mass relative to placebo, reinforcing previous studies showing a preservative effect on lean mass with GLP‐1 use.

### 3.4 Nutritional Effects

Nutritional outcomes were not reported explicitly, as most trials focused on the medication's safety and tolerability. However, several studies indirectly evaluated nutritional impacts through symptoms and adverse events/gastrointestinal events. Within the trials, nausea, early satiety and gastrointestinal discomfort may have contributed to reduced caloric intake, although these outcomes were not quantified as nutritional endpoints. In studies using hypocaloric diets, adherence was similar between placebo and GLP‐1 groups, indicating that gastrointestinal symptoms did not impair compliance with dietary guidance [29].

In the large trial by Pi‐Sunyer et al. [33], dietary advice was standardised. Despite significant gastrointestinal symptoms, participants maintained the prescribed caloric deficit, suggesting that drug‐induced anorexia did not compromise nutritional counselling efforts. While nausea and vomiting could theoretically reduce food intake, no study reported clinically significant nutritional deficiencies or adverse consequences related to micronutrient status.

### 3.5 Results of Risk of Bias Assessment

**FIGURE 2:**
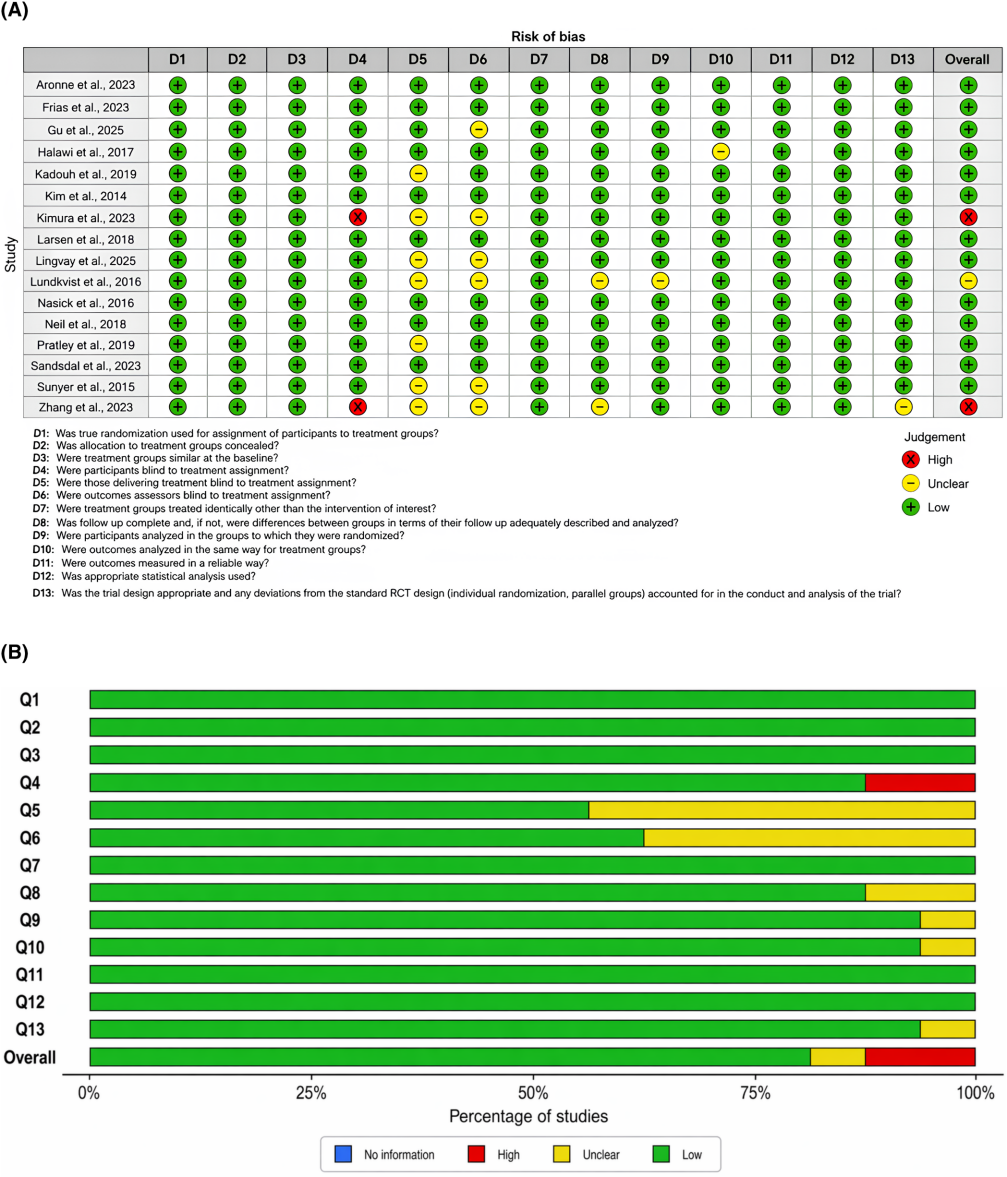
(A) Risk of bias represented by each item assessed and the distribution of risk of bias across the studies included in the systematic review. (B) Distribution of risk of bias across the assessed domains for all included studies.

Across the 16 studies included in this review, the majority demonstrated a low risk of bias. Thirteen studies (81.25%) were judged as having a low overall risk of bias, while two studies (12.5%) presented a high risk, and one study (6.25%) was rated as having an unclear risk of bias (Figure 2A,B).

The domains most frequently classified as “Unclear” were those related to blinding and reporting procedures. Specifically, Q5 (blinding of participants and personnel) and Q6 (blinding of outcome assessors) showed higher proportions of unclear assessments, reflecting limited methodological information in several publications. Q8 (completeness of follow‐up) also displayed occasional uncertainties, mainly due to insufficient reporting of attrition details (Table 2).

**TABLE 2:** Characteristics of randomised controlled trials assessing GLP‐1 and dual GIP/GLP‐1 agonists with lifestyle interventions.

Author	Study design	Sample characteristics	GLP‐1 agonist and placebo	Food intervention characteristics	Assessed outcomes	Main outcomes
Aronne et al. [25]	RCT double blind, 88 weeks	*n* = 670 Adults 18–75 years BMI ≥ 30 kg/m^2^ or ≥ 27 kg/m^2^ + ≥ 1 weight‐related comorbidity Without Diabetes	T: Tirzepatide (dual GIP/GLP‐1 agonist) 10 or 15 mg, weekly P: Placebo	Low‐calorie diet (−500 kcal/day) + supervised physical activity ≥ 150 min/week.	GE: Nausea, vomiting, diarrhoea, constipation.	Initial: GE: (T) 81.0% Nausea: 35.5%, Diarrhoea 21.1%, Constipation 20.7%, Vomiting 16.3%; Double‐masked period: Adverse events: (T) 60.3% (P) 55.8%
Frias et al. [26]	RCT double blind, 26 weeks	*n* = 352 Adults > 18 years T2D	D: Dulaglutide (1.5 mg weekly) O: Orforglipron (daily doses of 3, 12, 24, 36, or 45 mg) P: Placebo	Guidance on healthy eating, physical activity, and recognition and management of hypoglycaemia.	GE: nausea; diarrhoea; vomiting; constipation; dyspepsia; belching	GE: (P) 18.2%, (D)34%, (O) 44.1% to 70.4%; Nausea: (P) 5.5%, (D)18%, (O) 23.5% to 37.5%; Diarrhoea: (P)7.3%, (D) 12%, (O) 5.9% to 29%; Vomiting: (P) 1.8%, (D) 8%, (O) 5.9% to 35.5%; Constipation: (P) 1.8%, (D) 0%, (O) 2.9% to 22.2%; Dyspepsia: (P) 3.6%, (D) 2%, (O) 5.9% to 11.8%; Belching: (P) 0%, (D) 2%, (O) 3.1% to 18.5%
Gu et al. [27]	RCT, double blind, 44 weeks	*n* = 300 Adults ≥ 18 years T2D with overweight/obesity	S: Semaglutide (2.4 mg weekly) P: Placebo	Diet with a calorie deficit of approximately 500 kcal/day + ≥ 150 min/week of physical activity	GE: Nausea, vomiting, diarrhoea, constipation	(S) 64.6%; (P) 33.3% Diarrhoea: (S) 28.2% (P) 6.7% Nausea (S) 20.5% (P) 4.8%
Halawi et al. [30]	RCT double blind, 16 weeks	*n* = 40 Adults 18–65 years BMI ≥ 27 kg/m^2^	L: Liraglutide +0.6 mg weekly up to 3 mg once daily; P: Placebo	Standardised dietary and behavioural counselling for weight loss therapy.	Nausea; abdominal pain; diarrhoea	Nausea: (L) 63.15% (P) 19%
Kadouh et al. [38]	RCT double blind, 16 weeks	*n* = 40 Adults 18–65 years BMI ≥ 27 kg/m^2^	L: Liraglutide daily with an additional 0.6 mg per week until 3.0 mg P: Placebo	Behavioural weight‐management manual and individual counselling with motivational interviewing at baseline and follow‐up visits.	Lean mass	Lean mass: (L) –1.3 kg (−1.75, −0.1) (P) –0.65 (−1.13, 1.13)
Kim et al. [31]	RCT, double‐masked, placebo‐controlled, 14 weeks	*n* = 49 Adults 40–70 years BMI 27–40 kg/m^2^, with prediabetes	L: Liraglutide (0.6, 1.2, and 1.8 mg) P: Placebo	Energy‐restricted diet (−500 kcal/day) with maintenance of baseline physical activity, accompanied by weekly nutrition consultations.	GE: Nausea, vomiting, diarrhoea, constipation.	Nausea: (L) 67% (P) 26%
Kimura et al. [36]	RCT, open label, 24 weeks	*n* = 107 Adults: ≥ 20 years T2D	D: Dulaglutide (0.75 mg/week) S: Semaglutide (0.25 mg/week to 0.5 mg and to 1.0 mg)	Diet and exercise guidance were provided at baseline, and participants were told to follow the plan at each visit.	GE: Nausea, constipation; diarrhoea; abdominal fullness	GE: (D)13.2% (S) 46.3%; Nausea: (D) 7.5%, (S) 37%; Constipation: (D)1.9%, (S) 13%; Diarrhoea: (D) 1.9%, (S) 3.7%; Fullness: (D) 1.9%, (S) 1.9%
Nexøe‐Larsen et al. [32]	RCT, double blind, 12 weeks	*n* = 52 Adults: 18–64 years BMI ≥ 27 kg/m^2^	L: Liraglutide +0.6 mg weekly up to 3 mg once daily P: Placebo	Five nutritional and physical‐activity counselling sessions, hypocaloric diet −500 kcal/day (50% CHO, 20% protein, 30% fat, ≤ 10% saturated).	GE: Nausea, vomiting, diarrhoea, constipation.	(L) 88.5% (P) 34.6%
Lingvay et al. [28]	RCT, double blind, 72 weeks +9 weeks of follow‐up	*n* = 512 Adults ≥ 18 years BMI ≥ 30 kg/m^2^ T2D	S: Semaglutide 7.2 and 2.2 mg weekly P: Placebo	Structured lifestyle counselling, with a low‐calorie diet (~500 kcal/day) and physical activity ≥ 150 min/week	GE: Nausea, vomiting, diarrhoea, constipation.	(S) 53.1% (7.2 mg) (S) 51.5% (2.4 mg) (P) 25.5%
Lundkvist et al. [37]	RCT double‐masked, 24 weeks	*n* = 50 Adults 18–70 years BMI: 30–45 kg/m^2^ Without Diabetes	D/E: Dapagliflozin 10 mg once‐daily + Exenatide 2 mg once‐weekly P: Placebo	Standard guidance on a balanced diet and physical activity	Body composition, GE.	GE: (D/E) 64.0% (P) 40.0% Nausea: D/E 28.0% (P) 12.0% Body composition: Lean mass—D/E ↔ P
Nauck et al. [35]	RCT double blind, 3 years	*n* = 301 Adults: ≥ 18 years T2D	A30: Albiglutide (1×/week 30 mg) A50: Albiglutide (1×/week 50 mg) P: Placebo	Standard dietary guidelines are provided before treatment begins and enforced at each visit to the study centre.	Diarrhoea, nausea, reflux; constipation; vomiting; dyspepsia	Diarrhoea: (P) 11.9% (A30) 9.9% (A50)13.1%; Nausea: (P) 7.9% (A30) 9.9% (A50) 9.1%; Reflux: (P) 2% (A30) 1% (A50) 4%; Constipation: (P) 3% (A30) 2% (A50) 3%; Vomiting: (P) 1% (A30) 3% (A50) 3%; Dyspepsia: (P) 3% (A30) 2% (A50) 1%
Neil et al. [29]	RCT double blind, 52 weeks	*n* = 342 Adults: > 18 years BMI > 30 kg/m^2^ Without Diabetes	S: Semaglutide (once daily with dose escalation every 4 weeks) L: Liraglutide (once daily with dose escalation every 4 weeks) P: Placebo	−500 kcal of TDEE and nutritional advice every 4 weeks	GE: nausea; diarrhoea; constipation; vomiting	↔ Dietary adherence between groups GE: (S) 62% to 82% (L) 75% and (P) 75%; Nausea: (S) 31% to 54% (L) 45% (P) 45%; Diarrhoea: (S) 19% to 38% (L) 28% (P) 28%; Constipation: (S) 13% to 28% (L) 23% and (P) 23%; Vomiting: (S) 8% to 23% (L) 11% (P) 11%
Pratley et al. [34]	RCT double blind, 20 weeks	*n* = 295 Adults: 18–65 years; BMI > 30 or > 27 + comorbidity	E4: Efpeglenatide once‐weekly (4 mg) E6: Efpeglenatide once‐weekly (6 mg) E2/6: Efpeglenatide every 2 weeks (6 mg) E2/8: Efpeglenatide every 2 weeks (8 mg) P: Placebo	Low‐calorie diet, −500 kcal/day	Nausea; vomiting; diarrhoea; dyspepsia; constipation	Nausea: (P) 18.3%, (E4) 54.2%, (E6) 59.3%, (E2/6) 47.5%, (E2/8) 62.1%; Vomiting: (P) 6.7%, (E4) 22.0%, (E6) 22.0%, (E2/6) 16.9%, (E2/8) 32.8%; Diarrhoea: (P) 20%, (E4) 23.7%, (E6) 20.3%, (E2/6) 25.4%, (E2/8) 27.6%; Dyspepsia: (P) 3.3%, (E4) 20.3%, (E6) 27.1%, (E2/6) 15.3%, (E2/8) 25.9%; Constipation: (P) 8.3%, (E4) 16.9%, (E6) 20.3%, (E2/6) 15.3%, (E2/8) 20.7%
Sandsdal et al. [23]	RCT double blind, placebo‐controlled, 1 year.	*n* = 195 Adults 18–65 years BMI 32–43 kg/m^2^ Without Diabetes	L: Liraglutide 3.0 mg/day P: Placebo Groups: (P) Placebo (E) Exercise (L) Liraglutide (C) Combination (exercise + liraglutide)	8 weeks of ~800 kcal/day (Cambridge Weight Plan). Moderate‐to‐vigorous exercise (minimum of 150 min/week of moderate‐intensity or 75 min/week of vigorous‐intensity aerobic physical activity or an equivalent combination of both). Comparator: placebo without exercise.	GE: nausea, diarrhoea, or vomiting	GE: (P) 45% (E) 65% (L) 86% (C) 71%
Pi‐Sunyer et al. [33]	RCT double blind, 56 weeks	*n* = 3723 Adults: > 18 years BMI > 30 or > 27 + dyslipidemia or hypertension	L: Liraglutide (0.6 mg and weekly increments of 0.6 mg up to 3 mg) P: Placebo	Standardised dietary advice in individual or group sessions, up to week 68. Diet (−500 kcal with 30% of fat, 20% protein, and 50% carbohydrate).	Nausea; Diarrhoea; Constipation; Vomiting; Dyspepsia; Abdominal pain; Gastroesophageal reflux	Nausea: (L) 40.2% (P) 14.7%; Diarrhoea: (L) 20.9% (P) 9.3%; Constipation: (L) 20% (P) 8.7%; Vomiting: (L) 16.3% (P) 4.1%; Dyspepsia: (L) 9.5% (P) 3.1%; Abdominal pain: (L) 5.2% (P) 3.5%; Gastroesophageal reflux: (L) 0% and (P) 0.2%
Zhang et al. [24]	RCT, open‐label, 6 months	*n* = 68 Adults: 18 to 45 years BMI ≥ 24 kg/m^2^ and PCOS	D: Dulaglutide (1.5 mg/week) + restriction R: Caloric restriction	Dietary consultation and personalised dietary information leaflets with guidance on portion sizes and specific recipes for a week. A 1000–1300 kcal per day diet for everyone.	Lean mass (% and kg); Patients with ≥ 1 gastrointestinal events; nausea, vomiting; diarrhoea; constipation; abdominal distension; abdominal pain; belching	Lean mass: ↔ D and R; > 1 Gastrointestinal events: (D) 37.14% (R) 0%; Nausea: (D) 22.86% (R) 0%; Vomiting: (D) 20% (R) 0%; Diarrhoea: D—0% R—0%; Constipation: (D) 11.43% (R) 0%; Distension: (D) 5.71% (R) 0%; Abdominal pain: (D) 2.86% (R) 0%; Belching: (D) 2.86% (R) 0%

*Note*: ↔ indicates no difference.

Abbreviations: BMI, body mass index; GE, gastrointestinal events; GIP, gastric inhibitory polypeptide; GLP‐1, glucagon‐like peptide‐1; PCOS, polycystic ovary syndrome; RCT, randomised controlled trial; SB, subcutaneous; T2D, type 2 diabetes; TDEE, total daily energy expenditure.

In contrast, several domains demonstrated strong methodological consistency. Q1 (random sequence generation), Q2 (allocation to treatment groups) and Q9 (selective outcome reporting) were predominantly rated as low risk across studies, indicating adequate methodological rigour and transparency in these aspects (Figure 2B).

## 4 Discussion

This review presents findings across three interrelated domains: gastrointestinal events, muscle preservation and nutritional and lifestyle aspects, from randomised controlled trials (RCTs) with GLP‐1 agonists and dual GLP‐1 agonists. The results reveal a pattern of frequent gastrointestinal side effects, probable preservation of lean mass and no clear evidence of nutritional impairment after agonist use. Importantly, the included studies were predominantly conducted in the context of background lifestyle and dietary interventions; therefore, the findings should be interpreted as reflecting combined approaches rather than pharmacological effects in isolation.

Our results reinforce that gastrointestinal adverse events (GEs) remain the most common and clinically relevant limitation of GLP‐1/dual agonist therapy. Consistent with our observations, a recent meta‐analysis of 48 RCTs involving 27 729 participants reported an incidence of GEs of 11.7%, with nausea as the most frequent symptom. Among the agents, tirzepatide had the highest risk of nausea and diarrhoea, while dulaglutide and lixisenatide had the lowest [39]. Similarly, a systematic review focused on obese, non‐diabetic populations treated with semaglutide or tirzepatide found that EG were ~1.86 times more frequent with these agents than with placebo; the relative risk was higher for tirzepatide than for semaglutide [40].

This highlights a higher frequency of gastrointestinal symptoms with tirzepatide compared with other GLP‐1 receptor agonists, as well as a clear dose–response relationship: higher doses were associated with more frequent esophagogastroduodenopathy (EG). This increased dose‐related risk is corroborated by the literature, which demonstrates that higher doses of tirzepatide, as well as other agonists, are associated with higher rates of nausea, diarrhoea and vomiting compared to placebo or other comparators [41, 42, 43, 44]. This indicates that the choice of agent and dosage is important for balancing treatment efficacy and tolerability.

When analysing data regarding the combination of lifestyle interventions, such as diet control or exercise, high rates of GEs persist. This suggests that lifestyle changes may not be sufficient to mitigate the gastrointestinal side effects associated with these medications. For example, in trials that provided nutritional interventions or supervised exercise, gastrointestinal symptoms remained elevated [23, 24]. This reinforces the notion, also corroborated by systematic reviews, that the profile of gastrointestinal symptoms is largely determined by the pharmacodynamics of the agonists and not by modifiable lifestyle factors [41, 45]. Nevertheless, clinical monitoring by healthcare professionals, such as physical educators and nutritionists, is important to prevent worsening of these effects, which would likely be detrimental to treatment adherence and could compromise nutritional status [15, 46]. This interpretation is consistent with evidence showing that GLP‐1 receptor agonists, when combined with lifestyle modification, result in significant improvements in body weight and cardiometabolic outcomes, reinforcing their role as adjunct therapies rather than standalone interventions [47].

In this review, studies assessing lean mass reported small reductions, generally proportional to total weight loss [24, 37, 38]. Although a slight decrease in lean mass was observed with liraglutide, dulaglutide or the dapagliflozin–exenatide combination, these differences were not significant compared to controls, which typically received similar background lifestyle interventions, suggesting relative preservation of muscle tissue even in the context of weight loss. In this scenario, nutritional guidance may have helped maintain muscle mass. It is well established that a well‐planned diet with adequate protein intake can mitigate lean mass loss during weight loss [48, 49, 50, 51]. Although not used in the included trials, recent evidence indicates that protein supplements may help individuals achieve the recommended intake of 1.2–2.0 g/kg/day, and that compounds such as creatine monohydrate and β‐hydroxy β‐methylbutyrate can enhance the maintenance of lean mass and muscle strength during weight loss (Brittany et al. 2025). Taken together, these findings underscore the need for trials that combine nutritional interventions with more detailed musculoskeletal assessments, particularly of strength, function and muscle quality, in patients treated with GLP‐1 and dual GIP/GLP‐1 receptor agonists.

Although the studies did not directly assess diet quality or nutritional status, the results indicate that maintaining adequate food intake was possible despite adverse gastrointestinal effects [29, 33]. These findings suggest that structured nutritional interventions may help mitigate potential changes in food intake associated with GLP‐1 or GIP/GLP‐1 agonist therapy. Recent guidelines from international nutrition and obesity societies, including the American College of Lifestyle Medicine, the American Society for Nutrition, the Obesity Medicine Association and the Obesity Society, recommend that the use of these agonists be accompanied by structured nutritional monitoring, including assessment of food intake, prevention of nutritional deficiencies and behavioural support to promote dietary adherence. These recommendations indicate that pharmacological therapy alone may be insufficient to maintain nutritional adequacy, underscoring the importance of planned, supervised dietary interventions by qualified professionals [15].

**FIGURE 3:**
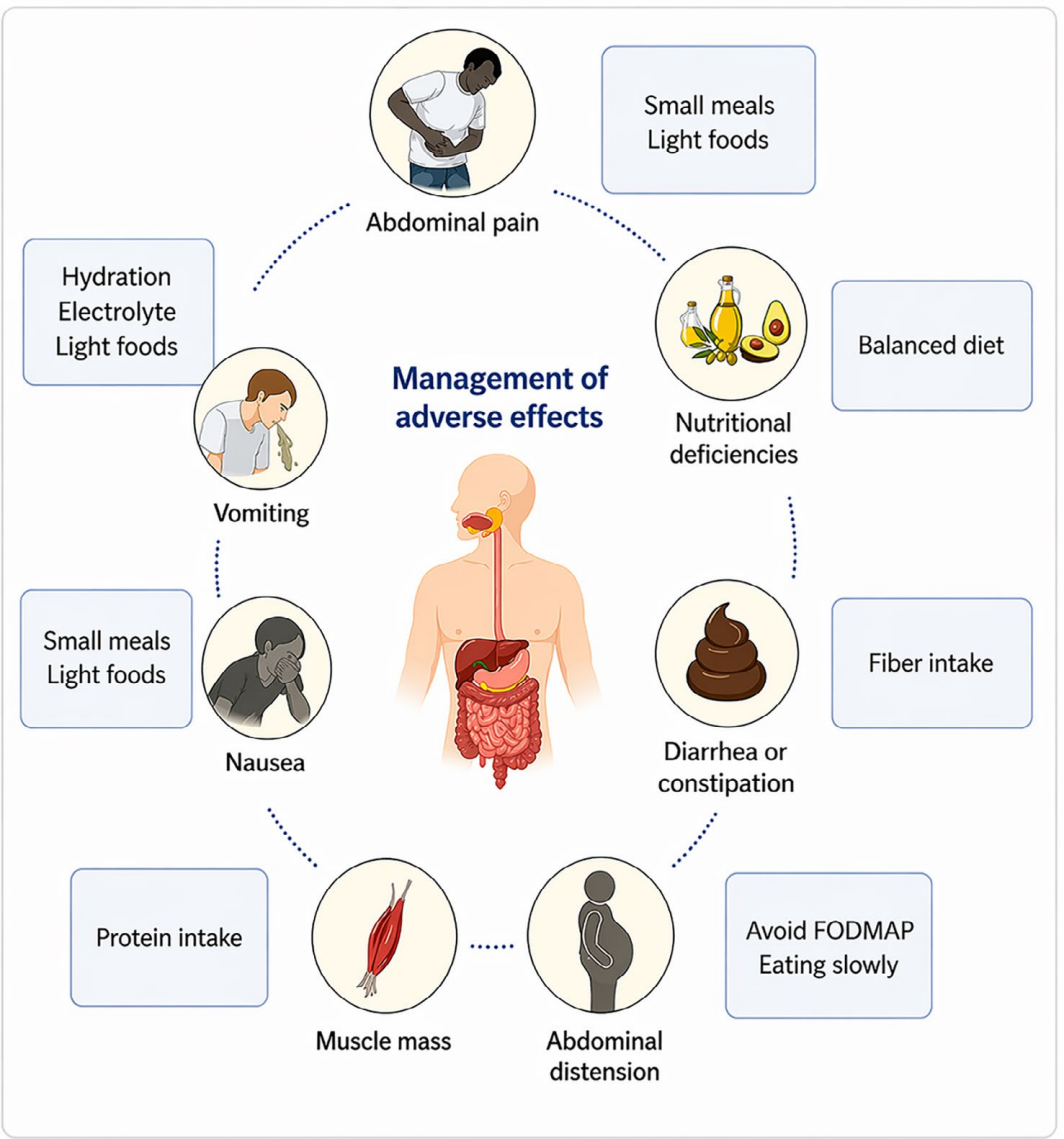
Nutritional management of adverse events during treatment with GLP‐1 and dual GIP/GLP‐1 receptor agonists.

The articles included in this review do not detail the main nutritional strategies and guidelines employed. However, dietary interventions described in the literature, initially proposed to manage gastrointestinal symptoms, may also help minimise adverse effects frequently observed with GLP‐1 and dual GIP/GLP‐1 agonists. Nausea may be alleviated by consuming small, frequent meals and selecting easily digestible foods [52, 53]. In cases of vomiting or diarrhoea, maintaining adequate hydration and electrolyte balance is essential, and consuming light, easily digestible foods also may aid recovery [52, 54, 55]. For constipation and diarrhoea, adequate fibre intake can support regular bowel habits [56]. Abdominal distension may be reduced by avoiding gas‐producing foods and eating slowly, whereas abdominal pain is typically managed with small, light and mildly seasoned meals [57, 58]. In addition to gastrointestinal symptom management, muscle mass preservation may be supported by adequate protein intake distributed throughout the day, with supplementation provided as needed (Brittany et al. 2025; [59]). Finally, preventing nutritional deficiencies requires a balanced, varied diet, monitoring of micronutrients and appropriate supplementation when dietary intake is inadequate, as shown in Figure 3 [60].

This study has some limitations. The review aimed to evaluate the effects of dietary strategies and nutritional management on gastrointestinal symptoms, muscle mass, bone health, nutritional adequacy and nutritional deficiencies in individuals treated with GLP‐1 or GIP/GLP‐1 agonists within the context of background lifestyle and dietary interventions. However, no studies were found that investigated bone health in this setting. Furthermore, most of the included trials did not perform statistical analyses of the reported adverse effects. In addition, the available evidence is largely derived from trials designed to assess pharmacological efficacy in combination with standardised lifestyle interventions, which limits the ability to isolate the independent effects of specific nutritional strategies. Also noteworthy is the absence of trials that directly compare the effectiveness of nutritional strategies with a control group, both of which received therapy with GLP‐1 or GIP/GLP‐1 agonists.

On the other hand, this study has several strengths. It is the first review to systematically address this topic, following PRISMA guidelines and registered in PROSPERO. In addition, the studies included in this review present a low risk of bias, are mostly double blind and involve a relevant sample size.

## 5 Conclusion

This systematic review shows that treatment with GLP‐1 receptor agonists and dual GIP/GLP‐1 agonists in the context of background lifestyle and dietary interventions requires an individualised approach. Within this context, structured nutritional guidance emerges as a strategic component to optimise care and support treatment adherence. Gastrointestinal events remain the main clinical limitation of these drugs, which aligns with their pharmacodynamic profile, but simple dietary adjustments can often help improve tolerability.

Findings on body composition suggest a relative preservation of lean mass, although lean mass tends to decrease as overall weight loss progresses. This highlights the importance of nutritional follow‐up to ensure adequate nutrient intake and to help maintain lean mass throughout treatment. Importantly, because current evidence derives from studies evaluating pharmacological therapies as adjuncts to lifestyle interventions, these findings should be interpreted within the context of combined treatment approaches rather than as isolated effects of pharmacotherapy or nutrition.

Despite these insights, important gaps remain and warrant rigorous investigation. Randomised clinical trials are needed to determine the optimal protein intake for maintaining muscle mass and function, as well as studies exploring potential effects on bone health. Generating such evidence is essential for establishing precise, fully evidence‐based dietary recommendations for patients undergoing treatment with GLP‐1 and GIP/GLP‐1 agonists.

## Funding

This work was supported by the Coordenação de Aperfeiçoamento de Pessoal de Nível Superior.

## Conflicts of Interest

The authors declare no conflicts of interest.

## Data Availability

Data sharing not applicable to this article as no datasets were generated or analysed during the current study.
